# A foresight whole systems obesity classification for the English UK biobank cohort

**DOI:** 10.1186/s12889-022-12650-x

**Published:** 2022-02-18

**Authors:** Stephen Clark, Nik Lomax, Mark Birkin, Michelle Morris

**Affiliations:** 1 Consumer Data Research Centre and School of Geography, University of Leeds, LEEDS, LS2 9JT UK; 2 School of Geography and Consumer Data Research Centre, University of Leeds, LEEDS, LS2 9JT UK; 3 School of Medicine and Consumer Data Research Centre, University of Leeds, LEEDS, UK

**Keywords:** Overweight, Obesity, Whole systems, Classification, UK biobank, K-means, Variable selection

## Abstract

**Background:**

The number of people living with obesity or who are overweight presents a global challenge, and the development of effective interventions is hampered by a lack of research which takes a joined up, whole system, approach that considers multiple elements of the complex obesity system together. We need to better understand the collective characteristics and behaviours of those who are overweight or have obesity and how these differ from those who maintain a healthy weight.

**Methods:**

Using the UK Biobank cohort we develop an obesity classification system using k-means clustering. Variable selection from the UK Biobank cohort is informed by the Foresight obesity system map across key domains (Societal Influences, Individual Psychology, Individual Physiology, Individual Physical Activity, Physical Activity Environment).

**Results:**

Our classification identifies eight groups of people, similar in respect to their exposure to known drivers of obesity: ‘Younger, urban hard-pressed’, ‘Comfortable, fit families’, ‘Healthy, active and retirees’, ‘Content, rural and retirees’, ‘Comfortable professionals’, ‘Stressed and not in work’, ‘Deprived with less healthy lifestyles’ and ‘Active manual workers’. Pen portraits are developed to describe the characteristics of these different groups. Multinomial logistic regression is used to demonstrate that the classification can effectively detect groups of individuals more likely to be living with overweight or obesity. The group identified as ‘Comfortable, fit families’ are observed to have a higher proportion of healthy weight, while three groups have increased relative risk of being overweight or having obesity: ‘Active manual workers’, ‘Stressed and not in work’ and ‘Deprived with less healthy lifestyles’.

**Conclusions:**

This paper presents the first study of UK Biobank participants to adopt this obesity system approach to characterising participants. It provides an innovative new approach to better understand the complex drivers of obesity which has the potential to produce meaningful tools for policy makers to better target interventions across the whole system to reduce overweight and obesity.

**Supplementary Information:**

The online version contains supplementary material available at 10.1186/s12889-022-12650-x.

## Background

Obesity presents a global challenge for society, with 650 million people, (13% of the total adult population), estimated as being obese worldwide [1, 2] with an additional 39% of adults being classed as overweight. This complex problem that involves a multitude of conflicting stakeholders, lifestyle choices, and physiological factors is not limited to adults, with there being over 340 million (18%) children and adolescents (aged 5–19 years) who are overweight or have obesity globally in 2016 [1]. Overweight and obesity prevalence continues to increase, and with it related comorbidities, in spite of the fact that it is preventable. In recent years there has been significant investment by United Kingdom (UK) research funders [3, 4] to better understand, and subsequently prevent, weight related health problems, however, overweight and obesity still prevail.

Research to date concludes that the drivers of obesity are complex and multifaceted, and not as simple as consuming less food and drink or moving more [5, 6]. Biological, social/cultural, ecological and psychological factors combine to create a tangled web of obesity promoting behaviours and environments [7]. In turn interventions and policy decisions to prevent overweight and obesity are complex. Success will inevitably be limited through tackling individual elements, such as proximity to fast food outlets [8] or green spaces [9], in isolation. Interventions leading to the greatest overweight and obesity prevention are hypothesised to come from a whole systems approach at a macro level – tackling multiple components of the obesity system at a large geographic scale [5, 10]. In the UK, ranked the 6th most obese OECD country [11], the obesity system was comprehensively mapped in 2007 as part of the UK government’s Foresight initiative [12, 13], yet attempts to utilise this, in its entirety, in obesity prevention have been limited [5, 14–16]. In a recent systematic review just thirty qualitative studies were identified as having taken a whole systems approach to obesity or other complex public health challenges [16]. More research took a quantitative approach, with 44 studies identified. These studies followed a range of study designs [17–19], but in the most part did not report their methods clearly, which given the complexity associated with a whole systems approach, makes interpreting the findings difficult. Linked data on all elements of the whole system are not readily available at an individual level, nor at the population level, further adding to the challenge and complexity associated with taking a whole systems approach to obesity (or other complex public health challenges) [15, 16, 18]. A comprehensive data mapping exercise against the Foresight obesity system map was completed in 2018, concluding that more can be done using traditional and novel data sources to incorporate more aspects of the obesity system into ongoing research [15].

We believe that a classification developed from the robust Foresight obesity system map framework, applied to a cohort with a range of weight statuses is novel and will demonstrate an approach to whole systems obesity research using existing cohort data. This will generate substantial transferrable utility of the methods reported here to other settings where it is valuable to predict obesity risk from wide ranging social data.

The aims of this study are to (i) investigate the feasibility of using the Foresight map as a framework for data driven obesity research and policy making, (ii) develop an obesity classification system where variable selection is informed by the Foresight system obesity map, applied to a sizeable sub-sample of the UK Biobank cohort of 500,000 adults, and (iii) test this against overweight and obesity outcomes. We hypothesise that distinct classes/clusters will be generated that differentiate different weight statuses. This method can then be applied to other cohorts and populations where identifying individuals at risk of, or already living with overweight or obesity is important to support prevention and healthier lifestyle behaviours.

## Methods

The UK Biobank is a large prospective cohort study of the 40 to 70 year old population, with baseline data collected between 2006 and 2010 in England, Scotland and Wales [20, 21]. The data were collected during an initial assessment at 22 regional centres, and provides information on the participants’ socio-demographic and economic situation, various physiological measurements, cognitive abilities and a limited number of biomarkers. Participants were asked to consent to the linking of their health episode and death data. Subsequently, sub-samples of the participants were invited back for repeat-assessments, for example to contribute to an imaging study or to measure physical activity levels using accelerometers. The cohort employed a range of data collection methods, including self-reported dietary surveys and Body Mass Index (BMI) generated from height and weight measured at the initial assessment visit.

**Fig. 1:**
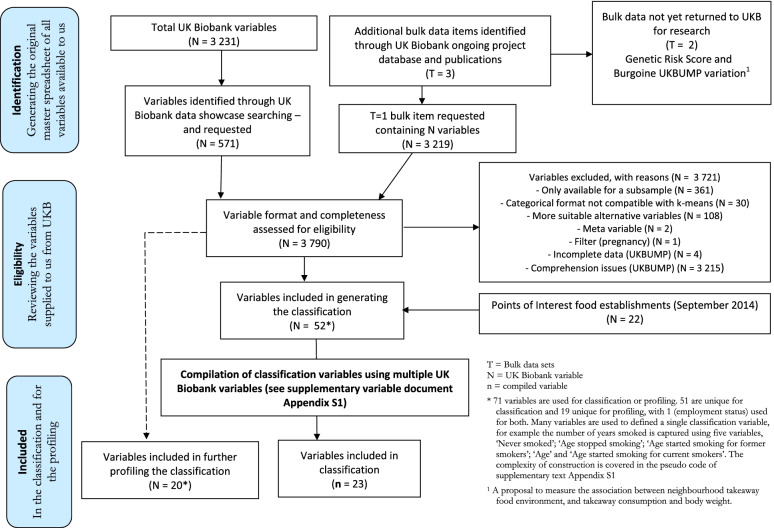
Modified PRISMA flow diagram for variable selection into Foresight informed k-means obesity classification of the UK Biobank cohort Genetic Risk Score [24]

In order to produce a classification of participants that aligns to known drivers for obesity, we select UK Biobank variables [22] that map onto the Foresight obesity systems map [5, 23]. This involved two ‘sifting’ exercises. The first sift was undertaken by three researchers (a population geographer, a nutritionist and a statistician) and involved the independent consideration of every UK Biobank variable then available and using the UK Biobank ‘Data Showcase’ to evaluate its utility when mapped to the Foresight obesity map. These three considerations were then discussed and a candidate sub-set of UK Biobank variables were requested. Having obtained these variables from UK Biobank a second sift was conducted by the same team. Access to the variables at the participant level allowed a more nuanced consideration of distributions and cross tabulations to further evaluate the utility of a sub-set of variables. Also at this point it became clear that many of the candidate variables had only partial coverage of UK Biobank participants (e.g. measurement from wearable accelerometers (*n* = 103,695) and food intake diaries (*n* = 70,714)). If such variables were to be included, the classification sample would be much reduced. This process is summarised in Fig. 1.

For analysis k-means classification is used to derive the obesity classification [25]. Classification methods attempt to group together participants that are most similar on a number of variables that describe their characteristics or nature, so that participants in each class will be more similar to their fellow class members than those in other classes.

In this study this classification was carried out using the k-means algorithm. K-means is a flexible and efficient algorithm, capable of handling the large volume of observations present in our data set. Other techniques are available e.g. hierarchical clustering or mixture models such as Latent Class/Profile Analysis [26] but these can be computationally heavy and require large memory storage when working with large datasets.

For k-means to be successfully applied it is necessary that the variables are not skewed, so that symmetric classes can form, and that each variable used is measured on a similar scale, so that each contributes a similar weight to the classification process, achieved here by converting each variable to a z-score. To correct for skewness Tukey’s ladder of power approach is used [27]. Prior to classification, the correlation between variables is measured to assess whether there is any potential redundancy in the variables where two or more variables are essentially capturing the same dimension.

To determine the number of classes, scree plots of the within classes sum of squares are custom and practice with k-means and additionally the reductions in this value from 1 to 12 classes are used. Another metric is that the resultant class sizes should be similar in nature, with no classes containing a particular high or small proportion of participants. For k-means analysis there can be no incomplete cases, therefore where information is either not known or not supplied by the participant, they are not used. Given that k-means clustering can only use continuous or integer count variables, we selected these for inclusion in the classification, and in addition the categorical variables with a match to the obesity systems map are used to profile the new classes.

To gain an understanding of the nature of each class, the centre of each class (which is essentially the mean value of each classification variable (which are given in Table 1) for the observations that are part of the class) is calculated. This helps to derive ‘pen portraits’ for each class – some classes will contain participants that, in aggregate, have high (or low) values for certain variables, and these variables provide the narrative for the pen portrait. Also, how the classes profile against other variables that have not been used to drive the classification are insightful. These additional variables come from UK Biobank and are also variables that are highlighted within the Foresight obesity map. The plausibility of these classes and their pen-portraits can help to validate the classification outcome.

**Table 1:** Variables used for classification

UK biobank variable	Foresight obesity variable	Foresight theme
Recreational PC use (hours)	1.04 Passive entertainment options	Societal Influences
TV watching (hours)	1.12 TV watching	
Smoking Duration (years)	1.16 Smoking cessation	
Household Size (people)	2.02 Face to face social interaction	Individual Psychology
Leisure and Social Activities (count)	2.02 Face to face social interaction	
Sleep Duration (hours)	2.04 Stress	
Stress (count)	2.04 Stress	
Metabolic Equivalent of Task (MET)	3.01 Physical activity	Individual Physical Activity
Mean Hand Grip Strength (Kg)^a^	3.02 Functional fitness	
Peak Expiratory Flow (litres/min)^a^	3.02 Functional fitness	
Time Spent Outdoors in Winter (hours)	3.04 Level of recreational activity	
Time Spent Outdoors in Summer (hours)	3.04 Level of recreational activity	
Vehicles per household member	4.11 Dominance of motorised transport	Physical Activity Environment
Percentage greenspace within 1000 m (%)	4.13 Walkability of living environment	
Pulse Rate (bpm)	5.02 Resting metabolic rate	Physiology
Townsend Deprivation Index (score)	6.01 Purchasing power	Food Production
Length of Working Week (hours)	6.06 Pressure on job performance	
Food establishments within 1000 m (count)	7.05 Food abundance	Food Consumption
Vegetable Consumption (tablespoon/day)	7.08 Food variety	
Fruit Consumption (pieces/day)	7.08 Food variety	
Low Fat Meat (% of meat consumption)	7.08 Food variety	
Alcohol (units of alcohol)	7.09 Alcohol consumption	
Age^b^	Outside Foresight	Outside Foresight

^a^These variables are bi-modal by gender. To correct for this each observation is standardised by the use of gender specific means and variances

^b^Age is not included in the Foresight systems map, but something we felt important to include since other studies have highlighted different obesity outcomes by age, through changes in food consumption [28] and activity patterns [29]

*NB* Node numbers initially reported in Morris, Wilkins [15]

Pen portrait names were assigned to the classes following a workshop with 35 multidisciplinary academics in attendance that aimed to generate meaningful names that were considered to be non-stigmatising.

In the results presented below, descriptive statistics are tabulated and multinomial regression models are estimated using cluster–robust standard errors, clustered by the assessment centre visited, to test whether the new classification can predict weight status. The utility of the multinomial regression is to test the ability of our classification to capture, in a statistically significant sense, whether membership of each of our classes is linked to a participant obesity status. In the regression, the relative risk ratios indicate risk of being overweight or obese compared to a healthy weight (with underweight omitted, *n* = 1699) and compared to the reference class ‘Younger, urban, hard-pressed’. An adjusted model is also estimated that additionally adjusts for gender, ethnicity, health, qualifications and employment status since these are variables that map onto the Foresight obesity map but due to their character are not included in the classification. To account for multiple testing, we adopted an alpha-level of 1% rather than 5% to judge the significance of the findings.

## Results

### Variable selection

Figure 1 presents results of UK Biobank variable mapping to the Foresight map, using a modified PRISMA flow chart where 23 variables are identified for inclusion in the classification and a further 20 variables used to profile the classification (see Appendix S1 for how these variables are constructed). The selection of 23 classification variables derived from UK Biobank variables are shown in Table 1.

After removing those participants that are pregnant, whose weight status is impacted by the pregnancy (371), those aged younger than 40 or older than 69, which was outside the recruitment criteria for the cohort (2431), and those that have missing data (154613) there are 345,091 participants available for classification. The Greenspace variable is only available for participants located in England [30, 31], meaning that participants living in Scotland and Wales are not part of this classification. The count of the food establishments within a straight line 1000 m distance was a bespoke variable calculated using the 1 km rounded up/down co-ordinates of the participants home location at the time of visiting the assessment centre and a database of Points of Interest [32]. Since we and others have found that the number of healthy and unhealthy food establishments in a neighbourhood are positively correlated, we did not differentiate by this characteristic [33, 34].

**Fig. 2:**
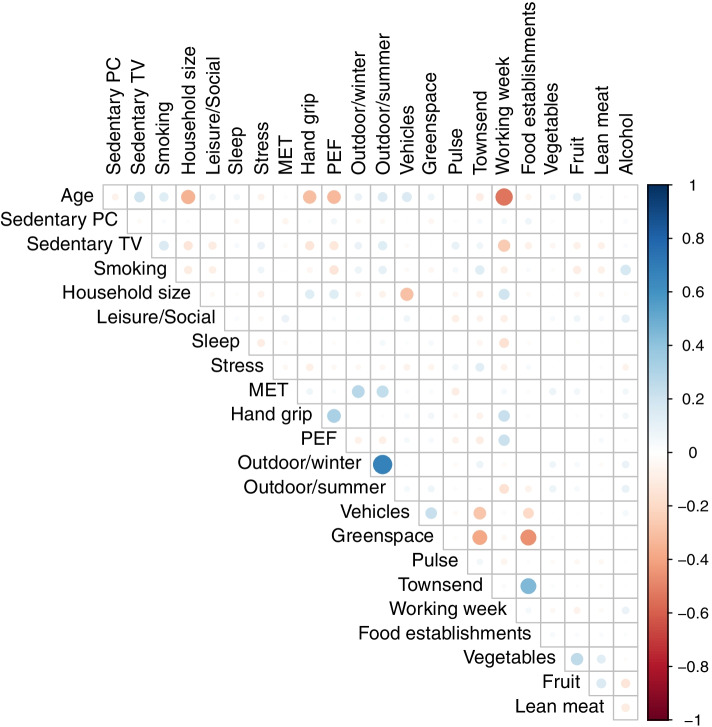
Heatmap of the correlation between classification variables

The distribution of key demographics for both the larger sample (excluding just those who are pregnant, younger and older) and the sub-sample used for classification is provided in Table 2 and shows that the sub-sample compares well with the full sample for most measures, with the exception of Townsend deprivation, where there is an indication that the sub-sample is less deprived and also there is no representation for Scotland or Wales. For this classification sample, none of the variables had a pairwise correlation greater than 0.7, as can be seen in the plot of the correlation matrix in Fig. 2, and as a result all the variables listed in Table 1 are used.

**Table 2:** Comparison of the characteristics of the full sample and the sub-sample used for classification

Variable	Category	Full sample	Classification sample
N		499,704	345,091
Gender	Male	45.6%	46.0%
	Female	54.4%	54.0%
Age	40–44	10.3%	10.6%
	45–49	13.2%	13.4%
	50–54	15.3%	15.4%
	55–59	18.2%	18.1%
	60–64	24.3%	24.3%
	65–69	18.7%	18.1%
Ethnicity	White	94.1%	94.8%
	Mixed	0.6%	0.6%
	Asian	2.3%	2.1%
	Black	1.6%	1.4%
	Other	0.9%	0.8%
	Not available	0.6%	0.3%
Townsend	Lowest 20%	20.0%	20.6%
Deprivation	20 to 40%	20.0%	20.9%
	40 to 60%	20.0%	20.4%
	60 to 80%	20.0%	19.9%
	Highest 20%	20.0%	18.1%
	Not available	0.1%	Not available
Assessment	Stockport (pilot)	0.8%	Not available
Region	North	41.4%	46.3%
	Midlands	15.7%	17.7%
	South	17.2%	20.5%
	London	13.7%	15.5%
	Wales	4.2%	Excluded
	Scotland	7.1%	Excluded
BMI	Underweight	0.5%	0.5%
	Healthy	32.3%	33.5%
	Overweight	42.2%	42.7%
	Having Obesity	24.3%	23.0%
	Not available	0.6%	0.2%

The profiling variables are shown in Table 3 and cover aspects such as the socio-demographic composition (e.g. gender and ethnicity), health status and illnesses (including specific morbidities), socio-economic composition (e.g. employment and occupation), the nature of their work (tasks involved and satisfaction) and geography.

**Table 3:** Variables used for profiling of the classification

UK biobank variable	Foresight obesity variable	Foresight theme
Education	1.1 Education	Societal Influences
Ethnicity	1.5 Sociocultural valuation of food	
Job involves shift work	2.4 Stress 2.4 Stress	Individual Psychology
Work satisfaction		
Diagnosed diabetes	2.10 Use of medicines and 5.12 Reliance on pharma remedies	Individual Psychology & Physiology
Diagnosed cancer		
Diagnosed other illness		
Overall health	3.2 Functional fitness 3.2 Functional fitness 3.2 Functional fitness	Individual Physical Activity
Limiting illnesses		
Breathless walking		
Job involves heavy lifting/physical tasks	3.6 Level of occupational activity	
Job involves walking/standing	4.12 Dominance of sedentary employment	Physical Activity Environment
BMI	5.24 Level of fat free mass	Physiology
Gender	6.10 Female Employment 6.15 Level of Employment 6.15 Level of Employment	Food Production
Employment		
Occupation		
Assessment Centre visited	Outside Foresight: Geographical location	Outside Foresight

### Cluster analysis

**Fig. 3:**
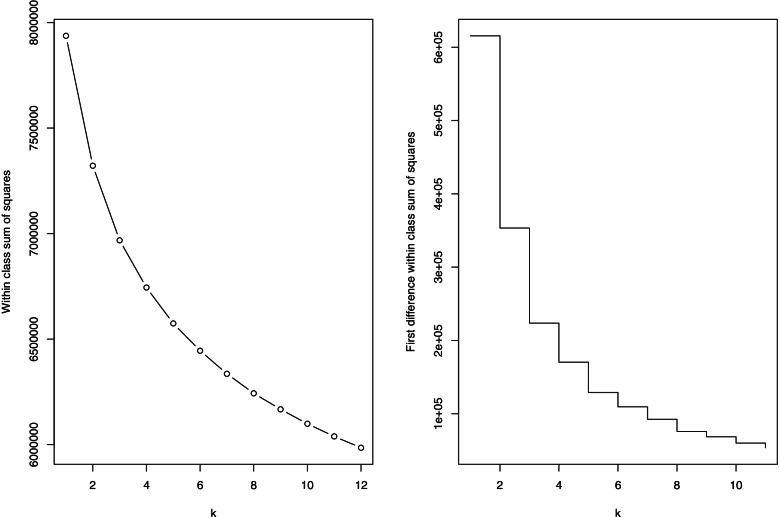
Scree plots of the within class sum of squares and its first difference for a range of classes

The left hand scree plot in Fig. 3 shows the within class sum of squares for various values of k, whilst the left hand plot show the reduction in this statistic as k increases. The left hand plot can be difficult to interpret, so attention will focus on the right had plot showing the reduction gained as k increases. These reductions are in effect the gradient in the scree plot and we are looking for value of k for which this changes. There are large reductions up to 5 classes, and from 5 through to 8 there are more modest reductions, with the reductions after 8 being smaller – to the extent that they could be consider linear. Here we have selected a generous 8 class solution that provides the largest scope for identifying a diverse range of classes, but are mindful that checks are required to ensure that there is sufficient differentiation in these 8 classes and that each class is not too small or large.

To this end, the classification centres and the number of participants in each class are shown in Table 4 with radial plots for the classifications available in supplementary Fig. S1. Using the characteristics of each classification group as described in Table 4 alongside the distribution of counts in various categories of profiling variables (which are not used in the classification) as shown in Supplementary Tables S1-S18 (with indicators of particularly high and low percentages relative to the percentage in the whole classification sample) a pen-portrait is constructed for each classification and presented in Table 5. These pen portraits indicate sufficient differentiation between the classes and even the smallest cluster is still large, representing nearly 8% of participants.

**Table 4:** Class centres on classification variables

	Younger, urban hard-pressed	Comfortable, fit families	Healthy, active and retirees	Content, rural and retirees	Comfortable professionals	Stressed and not in work	Deprived with less healthy lifestyles	Active manual workers	Full classification sample
Age	50.5	48.0	61.5	62.5	54.1	60.9	60.9	52.6	56.3
Recreational PC use (hours)	1.40	0.99	0.96	0.95	1.06	0.72	1.02	0.92	1.0
TV watching (hours)	1.92	2.06	2.60	2.82	2.20	3.77	3.86	2.81	2.7
Smoking Duration (years)	6.03	3.21	4.40	4.98	5.55	1.13	34.64	9.44	7.9
Household Size (people)	2.91	3.87	1.97	2.03	1.83	2.15	1.98	2.68	2.5
Leisure and Social Activities (count)	0.99	0.98	1.36	1.37	0.94	0.68	0.78	0.93	1.0
Sleep Duration (hours)	6.99	7.17	7.16	7.44	6.90	7.28	7.25	6.97	7.2
Stress (count)	2.69	2.25	2.55	2.13	2.58	2.99	2.89	2.29	2.5
Metabolic Equivalent of task (score)	8.38	9.09	13.24	11.81	6.84	4.74	7.54	22.04	9.9
Mean Hand Grip Strength^a^	0.18	0.58	−0.24	−0.05	0.19	−0.65	−0.32	0.36	0.0
Peak Expiratory Flow^1^	0.29	0.67	−0.23	− 0.01	0.29	− 0.53	− 0.55	0.18	0.0
Time Outdoors in Winter (hours)	1.33	1.21	2.47	2.18	0.95	1.45	2.00	5.37	1.9
Time Outdoors in Summer (hours)	2.57	2.77	4.55	4.64	2.15	3.24	4.23	7.43	3.7
Vehicles per household member	0.44	0.56	0.67	0.92	1.04	0.63	0.63	0.76	0.7
Greenspace within 1000 m (%)	22.6	53.9	31.2	65.1	51.7	42.6	40.7	46.0	45.5
Pulse Rate (bpm)	68.89	67.46	67.35	67.26	68.87	73.60	71.53	67.59	69.0
Townsend Deprivation (score)	8.11	3.57	6.07	3.00	4.07	5.21	6.32	5.40	5.0
Length of Working Week (hours)	31.9	33.2	8.5	5.1	37.9	7.1	9.5	37.4	20.8
Food estab. Within 1000 m (count)	157.8	18.1	71.5	10.2	22.6	34.2	46.7	33.7	47.6
Vegetable Consumption (tablespoon/day)	4.94	4.56	7.30	5.26	4.55	4.07	4.26	4.84	5.0
Fruit Consumption (pieces/day)	2.91	2.69	5.44	3.49	2.88	2.63	2.02	2.47	3.1
Low Fat Meat (propn meat consumption)	0.56	0.56	0.68	0.57	0.57	0.51	0.49	0.50	0.6
Alcohol (units)	14.10	13.66	9.91	15.66	15.38	6.62	21.90	23.45	14.6
N (%)	44,118 (13%)	54,439 (16%)	39,118 (11%)	56,428 (16%)	45,273 (13%)	41,982 (12%)	37,012 (11%)	26,721 (8%)	345,091

^a^These variables are bi-model by gender. To correct for this each observation is standardised by the use of gender specific means and variances

**Table 5:** Pen portraits for classification groups

**Younger, urban hard-pressed** (13% of classified participants)	
Participants in this class tend to be relatively younger than the full classification sample in UK Biobank, with an average age of 50.5 years. They also engage less with TV and use personal computers for recreational purposes the most. They live in a more urban setting, as typified by the low percentage of green space, the high number of food establishments close by and the low vehicle ownership. The neighbourhoods they live in are also the most deprived.	
**Comfortable, fit families** (16% of classified participants)	
These participants belong to the class with the youngest average age and the highest household size. They have good functional fitness, with high (standardised) hand grip strengths and peak expiratory flows. Satisfaction with health is relatively high and the proportion reporting excellent overall health is the highest of all classes. Stress levels are generally low, with participants reporting few stressful events and having a low pulse rate.	
**Healthy, active and retirees** (11% of classified participants)	
This is an older class of participants, having the second highest average age. The length of the working week is short, the proportion who are retired is higher than average and they take part in many leisure and social activities. Their diet is the healthiest, with high consumption of vegetables, fruit and lean meats. Those reporting excellent overall health is higher than average. They are located in neighbourhoods with low percentages of greenspace and many food establishments.	
**Content, rural and retirees** (16% of classified participants)	
A large proportion of these older participants are retired. They experience the least stress and have the longest sleep duration. They live in neighbourhoods with a high percentage of greenspace, are least deprived and have the fewest food establishments close by. Health satisfaction is higher than average, as is the proportion reporting overall excellent health. For those who work, job satisfaction is higher than average. This is the largest of the eight classes.	
**Comfortable professionals** (13% of classified participants)	
The participants in this class have the longest working week. Most are in employment and a large proportion are employed in managerial and professional occupations. While reported stress levels are about average, job satisfaction is lower than average. They also have the highest rate of household vehicle ownership, along with the smallest household size. They are the least likely to spend time outside during both summers and winters. This group live in the least deprived areas with higher than average green space.	
**Stressed and not in work** (12% of classified participants)	
This class of participants have the highest counts reporting stressful events and the highest pulse rate. A larger proportion than the sample average are looking after the home or family, are unable to work due to sickness/disability or are unemployed. Their function fitness is low, with low hand grip strength and peak expiratory flows and reported satisfaction with health is lower than average. This group watch a lot of TV but spend very little time on a personal computer. Their diet is relatively unhealthy with low consumption of vegetables and fruit, but alcohol consumption is low.	
**Deprived with less healthy lifestyles** (11% of classified participants)	
A distinctive feature of this class of participant is the number of years that they have been a smoker, by the far the highest of all classes. Their alcohol consumption is also high but their consumption of healthy food in the form of vegetables, fruit and lean meats are low. They live in neighbourhoods with moderate levels of deprivation. The proportion reporting being extremely or very happy with their health is lower than average. The proportion unable to work because of sickness or disability is relatively high, as is the proportion unemployed.	
**Active manual workers** (8% of classified participants)	
This final class are the most active, with a high metabolic equivalence score and many hours spent outside during the summer and winter. A large proportion of this group are male. The majority are employed with large representation in the skilled trades, process plant and machinery operatives and elementary occupations and jobs involve more walking, standing and physical tasks than seen in other groups. This group also have a long working week.	

Table 6 identifies a distinct profile for our classes by BMI category. Recall that BMI was not used during the classification exercise, yet the use of proxies for the drivers of obesity identified in the Foresight obesity system map have revealed differing weight status outcomes. The classes with the greatest proportion of people with a healthy weight are the ‘Younger, urban hard-pressed’ and the ‘Comfortable, fit families’ classes. Conversely the class with one of the lowest proportions in healthy weight are the ‘Deprived with less healthy lifestyles’. The class of ‘Active manual workers’ have high levels in the overweight category, and ‘Stressed and not in work’ and ‘Deprived with less healthy lifestyles’ have similar high proportions in the having obesity category.

**Table 6:** Distribution of classification by BMI category, median and mean

Weight status	Younger, urban hard-pressed	Comfortable, fit families	Healthy, active and retirees	Content, rural and retirees	Comfortable professionals	Stressed and not in work	Deprived with less healthy lifestyles	Active manual workers	Full classification sample
BMI Category									
Underweight	0.7%	0.5%	0.7%	0.3%	0.5%	0.6%	0.6%	0.1%	0.5%
Healthy	39.6%	39.6%	37.4%	34.9%	33.6%	27.5%	24.7%	24.6%	33.5%
Overweight	38.9%	41.7%	41.9%	46.8%	41.9%	40.1%	43.5%	48.2%	42.7%
Having obesity	20.7%	18.2%	19.8%	17.9%	23.9%	31.4%	30.8%	27.0%	23.0%
NA	0.2%	0.1%	0.2%	0.1%	0.1%	0.4%	0.3%	0.1%	0.2%
Summary BMI									
Median BMI	26.0	26.0	26.1	26.3	26.6	27.5	27.7	27.5	26.6
Mean BMI	26.8	26.6	26.8	26.8	27.3	28.3	28.3	28.0	27.3

A tabulation of the distribution of this classification by the region of the assessment centre attended in Table 7 also shows some interesting spatial patterns. London centres dominate the ‘Younger, urban hard-pressed’ class, with nearly 50% of those participants in this class attending a London centre, compared to less than 20% nationally. ‘Healthy, active and retirees’ also show a concentration in London. The more satisfied classes of ‘Comfortable, fit families’, ‘Content, rural and retirees’ and ‘Comfortable professionals’ are also spatially concentrated, having attended assessment centres in southern England. The midlands and northern assessment centres have a high concentration of the ‘Stressed and not in work’ participants, whilst the ‘Deprived with less healthy lifestyles’ are concentrated in the North East and North West of England. The ‘Active manual workers’ class has a fairly even split across the assessment centres, excepting London.

**Table 7:** Distribution of classification by location of assessment centre

Location of assessment centre	Younger, urban hard-pressed	Comfortable, fit families	Healthy, active and retirees	Content, rural and retirees	Comfortable professionals	Stressed and not in work	Deprived with less healthy lifestyles	Active manual workers	Full Classification Sample
North East England	6.0%	12.8%	11.0%	14.1%	12.7%	14.7%	14.7%	15.4%	12.6%
North West England	12.9%	16.0%	16.9%	15.8%	16.9%	18.3%	21.1%	18.9%	16.9%
Yorkshire	9.0%	19.0%	13.5%	20.2%	18.7%	17.6%	17.7%	18.1%	16.9%
Midlands	13.1%	17.2%	17.6%	18.6%	18.2%	20.3%	18.8%	18.5%	17.7%
Southern England	13.1%	28.0%	14.8%	27.2%	25.7%	16.4%	13.9%	18.4%	20.5%
London	45.9%	6.9%	26.3%	4.0%	7.8%	12.7%	13.9%	10.7%	15.5%

**Fig. 4:**
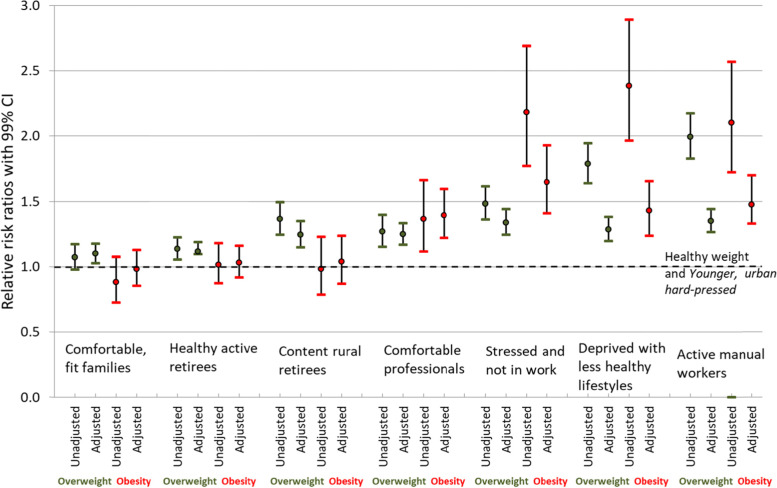
Multinomial logistic regression models to investigate the association between the whole system classes and weight status. The relative risk ratios indicate risk of being overweight or having obesity compared to a healthy weight (with underweight omitted) and compared to the reference class ‘Younger, urban, hard-pressed’. Adjusted models are adjusted for gender, ethnicity, health, qualifications and employment status

The estimates from the multinomial regression models shown in Fig. 4 identify that relative risk ratio for being overweight or having obesity compared to a healthy weight and the healthy UK Biobank class of ‘Younger, urban, hard-pressed’. For the overweight status these relative risk ratios are all significantly greater than 1.0, indicating that there is significant differentiation between our classes. This relative risk ratio is higher for classes: ‘Active manual workers’, ‘Stressed and not in work’ and ‘Deprived with less healthy lifestyles’. For those with a weight status of obese, the ‘Comfortable, fit families’, ‘Healthy active retirees’ and ‘Content rural retirees’ classes have a relative risk ratio that is not significantly different to that of healthy weight participants in the ‘Younger, urban, hard pressed’ class. A second model is estimated using some of the aforementioned profiling variables that are not used in the classification: gender, ethnicity, self-rated overall health, qualifications, and current employment status. In general, further adjustment with these additional profiling variables attenuates the relative risk ratios, especially for those whose weight status is generally not healthy.

## Discussion

This work is an important development that recognises the need for a whole systems approach to obesity research and policy making and incorporates this into the development of a classification to identify groups of individuals at highest risk of obesity - according to elements identified by the Foresight obesity systems map [5]. Cohort studies like the UK Biobank study capture a rich array of information on a large number of people, encompassing many of the seven Foresight themes: Societal influences, individual psychology, individual physical activity, physical activity environment, physiology, food production and food consumption.

Using the UK Biobank cohort study allowed us to evaluate our Foresight informed clusters to predict obesity using quality measures of our outcome of interest, overweight and obesity, where height and weight were measured, rather than self-reported.

To achieve our aims we have taken an existing large cohort of health and lifestyle data and attempted to map its data onto the Foresight obesity map. Whilst this process has identified some gaps in these data, this is perhaps to be expected given that the data is not bespoke for the obesity map and also the extensive nature of the obesity map, incorporating nearly 110 variables. However, the approach to variable selection as illustrated is, to the best of our knowledge, novel and has the potential to be replicated across other cohorts and data sources worldwide where quality overweight and obesity data may not be available, or where obesity related outcomes are of interest. A recent example of this can be seen in our study investigating whether an obesity classification can be used to identify individuals at risk of severe COVID-19 symptoms [35].

The classes we identified in this study highlight eight groups of people, similar in respect to their exposure to known drivers of obesity. The ‘Comfortable professionals’ class was most typical of the UK Biobank participant characteristics, and this extended to their BMI being average of the cohort. The classification also highlights three classes with increased relative risk of being overweight or having obesity: ‘Active manual workers’, ‘Stressed and not in work’ and ‘Deprived with less healthy lifestyles’.

On completion of the research the classification will be deposited back into the UK Biobank and made available to other researchers.

Social and environmental interventions present opportunities for change that can be implemented at both a micro and macro level, in communities and whole countries by local and national governments. However, it is not yet clear what the most effective approaches would be and how to synchronise changes across the system. In order to inform such change, we first need to better understand the collective characteristics and behaviours of people who are overweight or have obesity and how these differ from those who maintain a healthy weight. Traditionally, insights of this kind are generated from cohort studies that collect a wealth of data spanning individual behaviours, demographic characteristics, anthropometric measures and health related metrics. These encompass multiple areas of the obesity system, however they mostly relate to the individual, rather than wider environmental, societal and food system determinants. The classification we present here moves beyond using solely individual behaviours and incorporates systems drivers of behaviours enabling macro level insights into obesity risk.

A more holistic approach in obesity research has existed for some time, for example, clusters of lifestyle behaviours have been used in different settings to assist policy makers in targeting populations for a range of interventions. Often such clusters focus on individual elements of lifestyle, for example, dietary patterns [36, 37]. In some cases broader lifestyle clusters are identified [38, 39]. While insightful, data driven clusters of these kinds are not easily comparable against other cohorts and populations. Variables that drive the clusters are selected through a range of variable selection methods such as principal component or factor analysis, or more simply, by information that is available within the cohort. More generic clustering solutions such as geodemographic classifications, originally generated with marketing in mind, have been utilised by local government organisations and have demonstrated utility in highlighting groups with higher prevalence of obesity and related comorbidities [40–43]. Some geographic solutions have been tailored to specific application domain areas, for example CACI’s ACORN Wellbeing classification [44] which segments the population into four categories: Health Challenges; At Risk; Caution; Healthy - and further segregates these into 25 groups. Input data for such geodemographic classifications are largely derived from census data, open data or aggregated commercial sources. In research cohort studies rich data are available at an individual level, without the need to combine aggregated data sources using computational models to estimate patterns. Therefore, combining insight from geodemographic classifications with rich cohort data using a robust framework such as the Foresight system map has demonstrated here exciting possibilities for better understanding and leveraging the insight relevant to the whole obesity system.

The UK Biobank cohort is an example of an important study that is already contributing to obesity research, albeit focusing on specific areas of the obesity system, rather than the whole system. Using UK Biobank, [45] and [46] examine the influence of the availability of fast food on obesity. Activity levels are studied as commuting behaviour in two studies reported by Flint and Cummins [47] and Flint, Webb [48] and by an examination of how the built environment can influence activity patterns [9, 48]. Other potential influences for obesity have also been studied using UK Biobank participant data, including socio-economic factors [49], smoking [50], presence of morbidities [51], work patterns [52] and ethnicity [53]. In this research study, we incorporate all relevant drivers of obesity as identified by the theoretical framework presented in the Foresight obesity system map, therefore extending what has been done before using either individual or environmental influences upon obesity.

Green, Strong [54] conducted a classification exercise, based solely on data from participants classified as having obesity, meaning that the purpose of the classification was not to differentiate different weight statuses. Furthermore, this classification did not use the Foresight obesity system map to inform the classification development.

### Limitations

We found that Foresight themes relating to individual behaviours were better captured within the cohort data, with the environmental, societal and food production areas more difficult to populate. Given that the UK Biobank cohort was first established prior to the 2007 Foresight report, it is not surprising that they didn’t consider collecting information from participants about their exposures to aspects of the wider obesity system, especially so since the aims of the UK Biobank cohort study are broad in respect to helping to gain a better understanding of determinants of disease, not just obesity. The Foresight obesity systems map incorporates some upstream determinants of behaviours, for example, elements of food production in addition to food consumption [15]. With regards to food consumption, the quantity of this information was challenging to incorporate into the classification. This is not so much of an issue since we were not trying to generate dietary pattern clusters, instead we have generated whole systems obesity clusters so therefore to collapse the dietary information into a small group of food consumption variables is acceptable, a process explained in supplementary appendix S1. While some dietary information is lost using this approach, it achieved our goal of condensing the dietary information such that it did not dominate the classification. It is also important to acknowledge that dietary data collected in the UK Biobank was self-reported and therefore subject to bias. However, a recent validation study of the Oxford WebQ dietary questionnaire suggests reasonable agreement against biomarkers when compared with dietary recall interviews [55]. Other self-reported measures, such as TV watching were also used in our classification and we are not aware that these have undergone such scrutiny.

It was originally envisaged that more variables would be available to map onto the Physical Activity Environment Foresight theme from the UK Biobank Urban Morphometric Platform bulk data [56]. However, on receipt of these data we found that there were issues around the completeness and comprehension, for example: incomplete greenness data and inconsistency between outlet density and count metrics in the same field. Recognising that there was still a need to incorporate some linked environmental data that considers the context in which individual behaviours are conditioned, we used the alternative greenspace indicator of Wheeler [31] and calculated our own food environment exposure, recognising that positive correlations exist between counts of healthy and unhealthy food establishments [33, 34].

In developing the new classification we found that both age and gender were important with respect to the outcome BMI and also as a confounding factor with some of the classification variables – e.g. hand grip strength. Supplementary material in appendix S1 explains in detail how gender was used to standardise these variables. While these characteristics do not appear in the Foresight maps as features of the obesity system, they are still important to consider when developing methods to better understand obesity. This is suggestive that perhaps the Foresight obesity system map should in fact encompass more nodes within its complex system map.

This study employed a comprehensive variable selection process for the development of the obesity classification, driven by the Foresight obesity system map. This process was rigorous and completed by three independent researchers from a range of backgrounds and agreement on variable selection reached by informed discussions. However, there are limitations to this approach, especially with respect to the omission of categorical variables, which cannot easily be used in the k-means clustering algorithm. Other approaches do exist which can deal with categorical variables [57, 58], however all algorithms require decisions and compromises to be make, and k-means has proved efficient and effective in producing distinct classes that exhibit significant differences in both weight status and the profiling variables identified in Table 3.

Here we only considered baseline BMI, as the follow up measurements in the cohort were only for subsamples. We did investigate agreement in measurement between the multiple time points and found that the baseline information was a strong indicative measure of subsequent BMI for us to test our classification against. In the same vein, we did not make use of other candidate variables which were only available for a subset of UK Biobank participants, for example the accelerometer data.

Some caution must be taken when interpreting the results in this paper given that the UK Biobank participants are, by design, from an older demographic, which is largely White British in ethnicity, and is also not generalisable to the wider UK population [59]. These features mean that whilst obesity is seen to persist from childhood into adolescence, adulthood, middle age and into the senior years, it is only the later phase of this life course that is picked up here [60, 61]. That said, any biases in these data do not impact upon the methods and process we present for selecting variables and development of such a classification since k-means does not require that a sample be representative of a population in order to make certain inferences.

### Policy recommendations and future applications

We recommend using the Foresight obesity systems map as a framework to inform variable selection for obesity research and to drive policy making. We have demonstrated its ability to produce a clustering solution within the UK Biobank. Therefore, while classes derived from the clustering remain relevant to the population the data relate too, the methods for variable selection are consistent, meaning that methods can be reproduced for different data and compared. However, in order for this to be most effectively applied we would recommend a broader representation of the data from each of the Foresight themes, which may be achieved through a more targeted data collection in such cohort studies, or through collating other sources of information on the system, such as those provided by consumer data. A combination of existing data sources would present a powerful alternative to new primary data collection.

A further development of our classification, which would enrich understanding of areas and groups of people most in need of positive change, would be to incorporate a geographic identifier to the whole system classification, akin to geodemographic classifications such as the Output Area Classification from the Office for National Statistics [62] or commercial classifications like Cameo [63]. With geographic identifiers incorporated the potential use for this type of classification would extend to better targeting of resource and support, for example they could be used by national policy makers to allocate funds to areas most in need of making system wide changes and in turn by public health directorates in local authorities to allocate resources to neighbourhoods most requiring support.

Overweight and obesity have been substantial public health challenges for some time, but in light of the recent ‘call to action’ within the UK’s National Obesity Strategy, citing COVID-19 as a wakeup call, methods to better target resources to improve health through reducing prevalence overweight and obesity, are more important than ever [64]. Indeed, we have found that our classification reveals significant differences in exposure, treatment and mortality for COVID-19 by assessing outcomes in the linked test, hospitalisation and deaths data available within UK Biobank [35] .

## Conclusions

This work presents an innovative new approach to better understanding the whole systems drivers of obesity which has the potential to produce meaningful tools for policy makers to better target interventions across the whole system to reduce overweight and obesity.

## Supplementary Information

**Additional file 1.**

**Additional file 2.**

**Additional file 3.**

## Data Availability

The datasets generated and/or analysed during the current study are not publicly available due access arrangements required by UK Biobank. Please contact access@ukbiobank.ac.uk for details. Specifically, these data can be obtained by registering an interest (https://www.ukbiobank.ac.uk/enable-your-research/register) and submitting an application for data access (https://www.ukbiobank.ac.uk/enable-your-research/apply-for-access), quoting Project ID 30846. The software for the analysis is available from the corresponding author on reasonable request.

## Abbreviations

BMI: Body Mass Index
COVID-19: Coronavirus disease
OECD: Organisation for European Economic Co-operation
PRISMA: Preferred Reporting Items for Systematic Reviews and Meta-Analyses
UK: United Kingdom of Great Britain and Northern Ireland
UKB: UK biobank
