# Correction to: A foresight whole systems obesity classification for the English UK biobank cohort

**DOI:** 10.1186/s12889-022-13162-4

**Published:** 2022-04-11

**Authors:** Stephen Clark, Nik Lomax, Mark Birkin, Michelle Morris

**Affiliations:** 1 Consumer Data Research Centre and School of Geography, University of Leeds, LEEDS, LS2 9JT UK; 2 School of Geography and Consumer Data Research Centre, University of Leeds, LEEDS, LS2 9JT UK; 3 School of Medicine and Consumer Data Research Centre, University of Leeds, LEEDS, UK

**Correction to BMC Public Health 22, 349 (2022)**

**https://doi.org/10.1186/s12889-022-12650-x**

The original publication [1] of this article contained an incorrect supplementary file 3. The incorrect and correct supplementary file are available in this correction article, the updated information is shown in red. The original article has been updated with the correct supplementary file.

## Supplementary Information

**Additional file 1: Supplementary file 1.** Incorrect version of supplementary 3 as originally published.

**Additional file 2: Supplementary file 2.** updated version of supplementary 3.
