# Supplementary material for: A foresight whole systems obesity classification for the English UK biobank cohort
Source: BMC Public Health. 2022 Feb 18;22:349. doi: 10.1186/s12889-022-12650-x (PMC8856870; doi:10.1186/s12889-022-12650-x)
Supplement: Supplementary file 1 — Additional file 1. [file 12889_2022_12650_MOESM1_ESM.docx]

**Supplementary Appendix S1**

This note provides pseudo code to illustrate how each classification variable was constructed from a range of UK Biobank variables. The number after the character ‘f’ denotes the Data-Field number used in the UK Biobank Data showcase (<https://biobank.ndph.ox.ac.uk/ukb/>). The following _X_Y code denotes the baseline data (X=0) and the instance (Y).

**# Age**

date_of_attending_assessment_centre_f53_0_0-function(year_of_birth_f34_0_0,month_of_birth_f52_0_0)

**# Sedentary activity PC**

time_spent_using_computer_f1080_0_0

**# Sedentary activity TV**

time_spent_watching_television_tv_f1070_0_0

**# Smoking years**

Never smokers

0

Former smokers

age_stopped_smoking_f2897_0_0-age_started_smoking_in_former_smokers_f2867_0_0

Current smokers

age_0_0-age_started_smoking_in_current_smokers_f3436_0_0

**# Household size**

When type_of_accommodation_lived_in_f670_0_0 is NOT Sheltered accommodation or Care home then this is the number_in_household_f709_0_0

**# Social activities**

Count of:

leisuresocial_activities_f6160_0_?

Sports club or gym

Pub or social club

Religious group

Adult education class

Other group activity

**# Sleep duration**

sleep_duration_f1160_0_0

**# Stresses**

Count of:

sleeplessness_insomnia_f1200_0_0 +

worrier_anxious_feelings_f1980_0_0 +

loneliness_isolation_f2020_0_0 +

seen_doctor_gp_for_nerves_anxiety_tension_or_depression_f2090_0_0 +

seen_a_psychiatrist_for_nerves_anxiety_tension_or_depression_f2100_0_0 +

illness_injury_bereavement_stress_in_last_2_years_f6145_0_?

Serious illness, injury or assault to yourself

Serious illness, injury or assault of a close relative

Death of a close relative

Death of a spouse or partner

Marital separation/divorce

Financial difficulties

**# MET activity levels**

(duration_of_walks_f874_0_0*3.3+

duration_of_moderate_activity_f894_0_0*4.0+

duration_of_vigorous_activity_f914_0_0*8.0)/60

**# Hand grip**

(my_ukb_data$hand_grip_left_0_0+my_ukb_data$hand_grip_right_0_0)/2

**# Peak exploratory flow**

Ignore peak_expiratory_flow_pef_f3064_0_? less than 25

(peak_expiratory_flow_pef_f3064_0_1+

peak_expiratory_flow_pef_f3064_0_2+

peak_expiratory_flow_pef_f3064_0_3)/3

**# Outdoor activity in winter**

time_spent_outdoors_in_winter_f1060_0_0

**# Outdoor activity in summer**

time_spend_outdoors_in_summer_f1050_0_0

**# Vehicles per household member**

number_of_vehicles_in_household_f728_0_0/number_in_household_f709_0_0

**# Percentage greenspace within 1000m**

Greenspace_percentage_buffer_1000m_f24500_0_0

**# Pulse**

(pulse_rate_automated_reading_f102_0_0+pulse_rate_automated_reading_f102_0_1)/2

**# Townsend**

# Make wholly positive

townsend_deprivation_index_at_recruitment_f189_0_0+6.5

**# Working week**

length_of_working_week_for_main_job_f767_0_0

**# Count of food establishments within 1000m**

Counts of food establishments:

9470798 (Tea and coffee merchants)

1020043 (Restaurants)

1020034 (Pubs, Bars, Inns)

9470662 (Butchers)

9470665 (Delicatessens)

9470666 (Fishmongers)

9470667 (Frozen foods)

9470668 (Green and new age goods)

9470669 (Grocers, farm shops and pick your own)

9470670 (Herbs and spices)

9470672 (Organic, health, gourmet and kosher foods)

9470705 (Markets)

9470819 (Supermarket chains)

1020013 (Cafes, snack bars and tea rooms)

9470699 (Convenience stores)

1020025 (Internet cafes)

1020018 (Fast food and takeaway outlets)

1020019 (Fast food delivery services)

1020020 (Fish and chip shops)

9470661 (Bakeries)

7400524 (Baking and confectionery)

9470663 (Confectioners)

within a straight line distances of 1000m from

home_location_at_assessment_east_coordinate_rounded_f20074_0_0

home_location_at_assessment_north_coordinate_rounded_f20075_0_0

**# Healthy foods vegetables**

cooked_vegetable_intake_f1289_0_0+salad_raw_vegetable_intake_f1299_0_0

**# Healthy foods fruit**

fresh_fruit_intake_f1309_0_0+dried_fruit_intake_f1319_0_0

**# Lean meats**

Convert categories of consumption to daily

| Category | Annual amount |
| --- | --- |
| Never | 0 |
| Less than once per week | 18 |
| Once a week | 52 |
| 2-4 times a week | 156 |
| 5-6 times a week | 286 |
| Daily | 365 |

Lean_meat_0_0<-(oily_fish_0_0+fish_0_0+poultry0_0)/

(oily_fish_0_0+my_ukb_data$fish_LIDA_0_0+poultry_0_0+ process_meat_0_0+beef_0_0+lambmutton_0_0+pork_ _0_0)

**# Alcohol**

average_weekly_red_wine_intake_f1568_0_0*1.5+

average_weekly_champagne_plus_white_wine_intake_f1578_0_0*1.5+

average_weekly_beer_plus_cider_intake_f1588_0_0*2.9+

average_weekly_spirits_intake_f1598_0_0*1.0+

average_weekly_fortified_wine_intake_f1608_0_0*1.25
