# Supplementary material for: A foresight whole systems obesity classification for the English UK biobank cohort
Source: BMC Public Health. 2022 Feb 18;22:349. doi: 10.1186/s12889-022-12650-x (PMC8856870; doi:10.1186/s12889-022-12650-x)
Supplement: Supplementary file 2 — Additional file 2. [file 12889_2022_12650_MOESM2_ESM.docx]

**Classification radar plots**

The following charts show how distinctive each of the derived classification is.

There is one chart for each classification of participants (making 8 in total). Each classification variable is aggregated to a mean for all participants that are allocated to the classification. Where this mean is positioned on a normalised scale (with a mean of zero and unit variance) defined by all participant’s data is show on the chart. Thus a value greater than 0.0 means that the participants in that class tend to have a higher value for this variable than in the classification sample as a whole. The greater the value, the more distinctive. A value less than 0.0 means that the participants tend to have a lower value for this variable. The variables are sorted, clockwise, from the greatest positive standardised difference to the greatest negative.

Within each plot, the following labels relate to the classification variables used:

| **UK Biobank Variable** | **Plot label** |
| --- | --- |
| Recreational PC use | PC Use |
| TV watching | TV Watching |
| Smoking Duration | Smoking |
| Household Size | HHD Size |
| Leisure and Social Activities | Leisure/Social |
| Sleep Duration | Sleep |
| Stress | Stress |
| Metabolic Equivalent of Task | MET |
| Mean Hand Grip Strength | Hand Grip |
| Peak Expiratory Flow | PEF |
| Time Spent Outdoors in Winter | Winter |
| Time Spent Outdoors in Summer | Summer |
| Vehicles per household member | Vehicles |
| Percentage greenspace within 1000m | Greenness |
| Pulse Rate | Pulse |
| Townsend Deprivation Index | Townsend |
| Length of Working Week | Working Week |
| Food establishments within 1000m | Food PoI |
| Vegetable Consumption | Vegetables |
| Fruit Consumption | Fruit |
| Low Fat Meat | Meat |
| Alcohol | Alcohol |
| Age | Age |


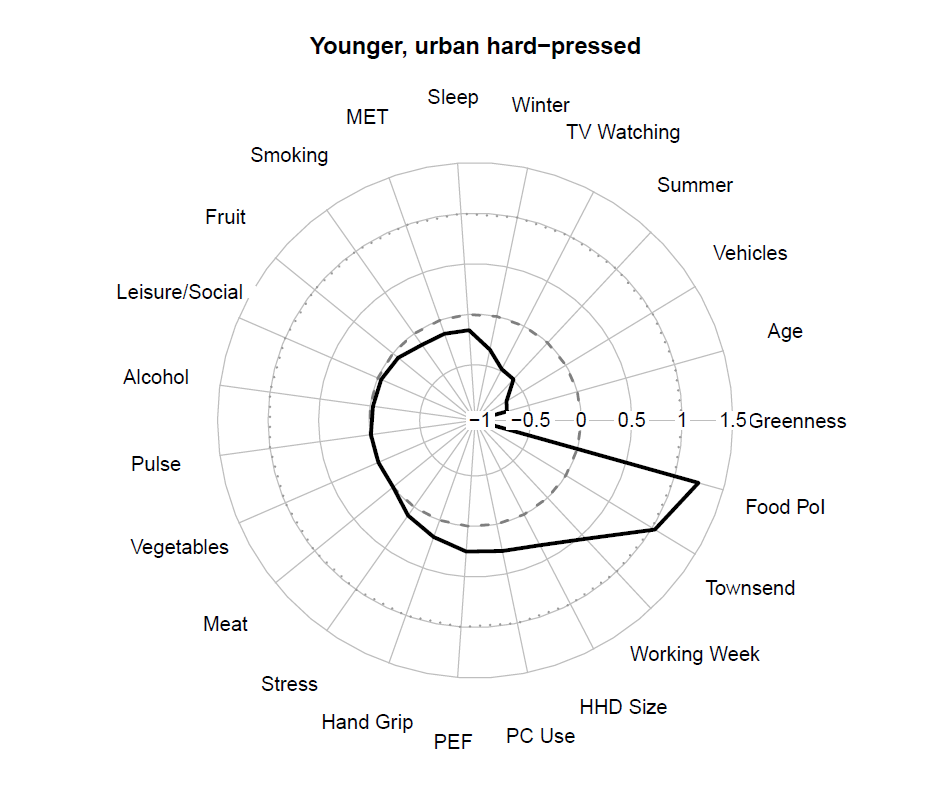


Figure S1a : Classification radar plots for the Younger, urban hard-pressed group


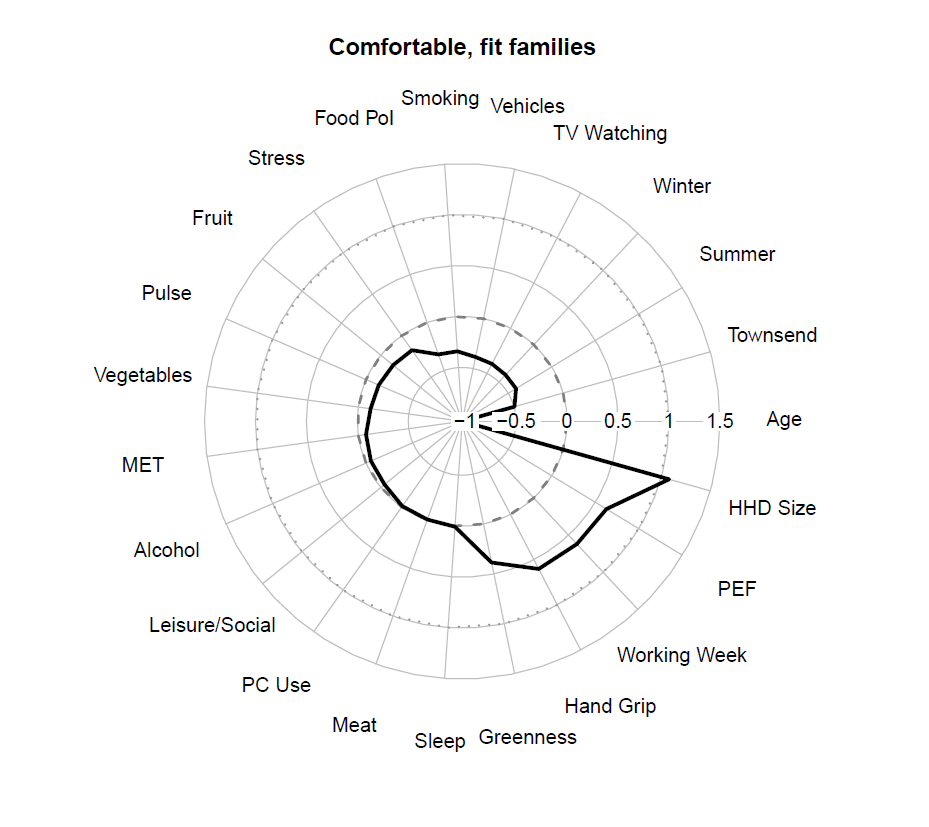


Figure S1b : Classification radar plots for the Comfortable, fit families group


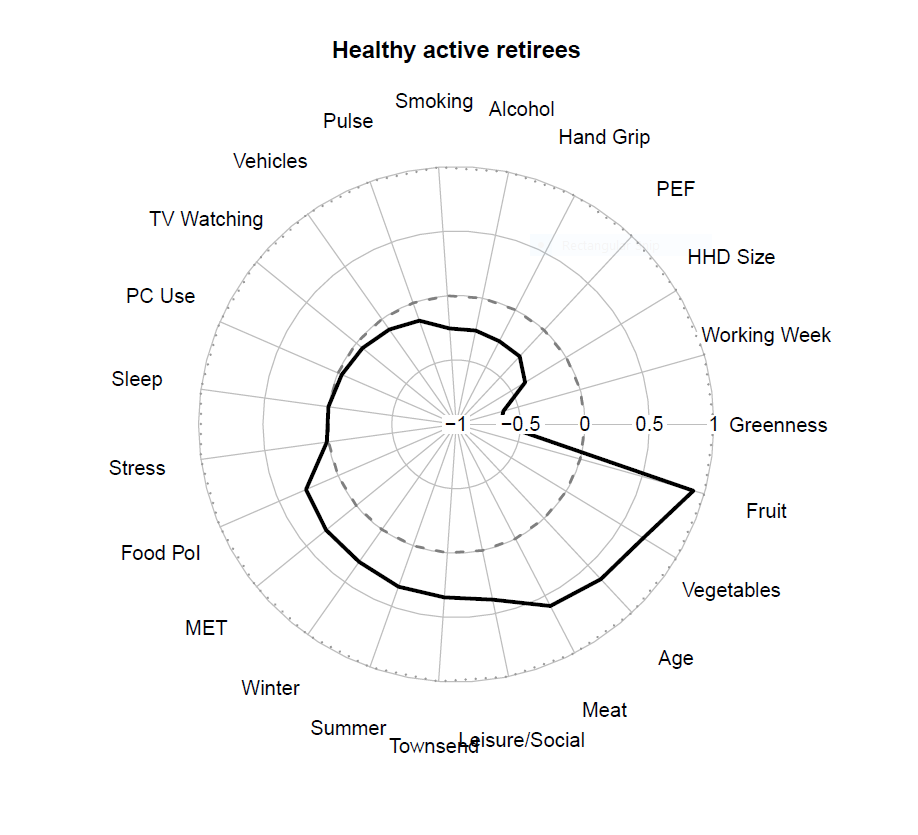


Figure S1c : Classification radar plots for the Healthy active retirees group


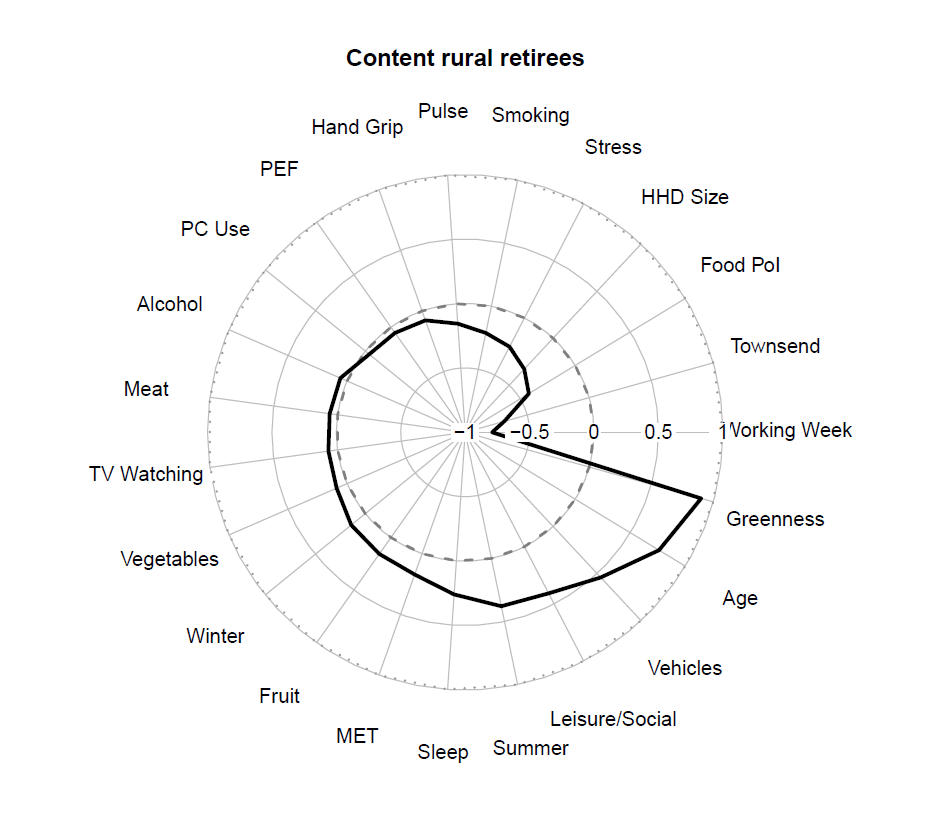


Figure S1d : Classification radar plots for the Content rural retirees group


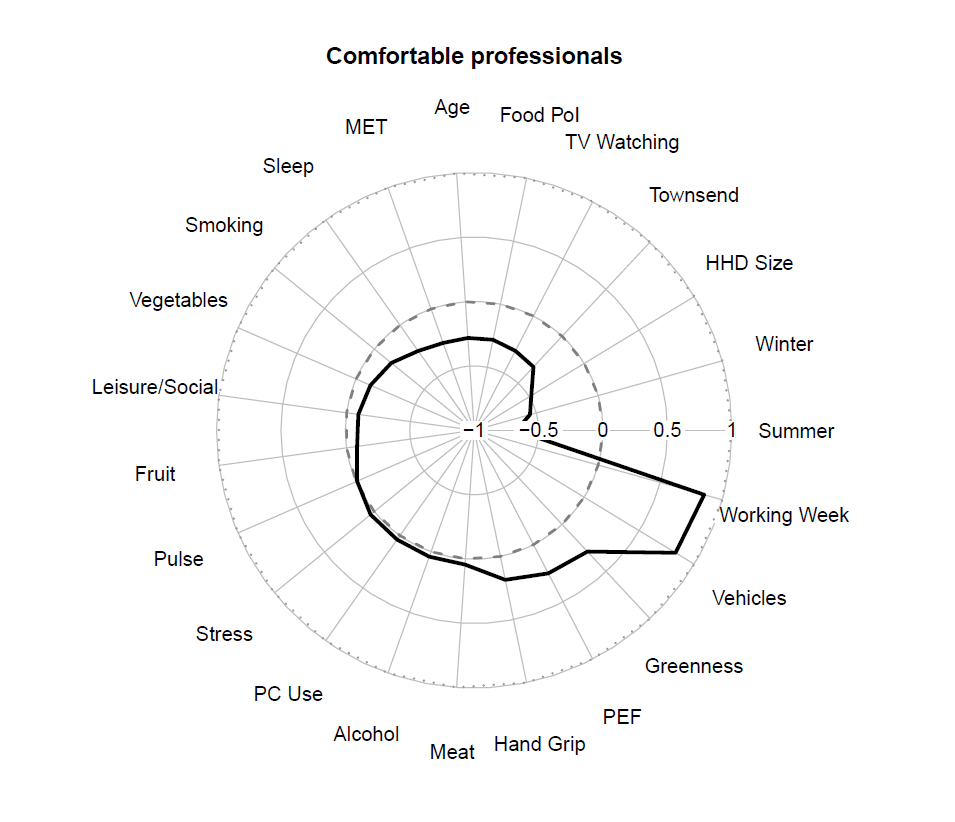


Figure S1e : Classification radar plots for the Comfortable professionals group


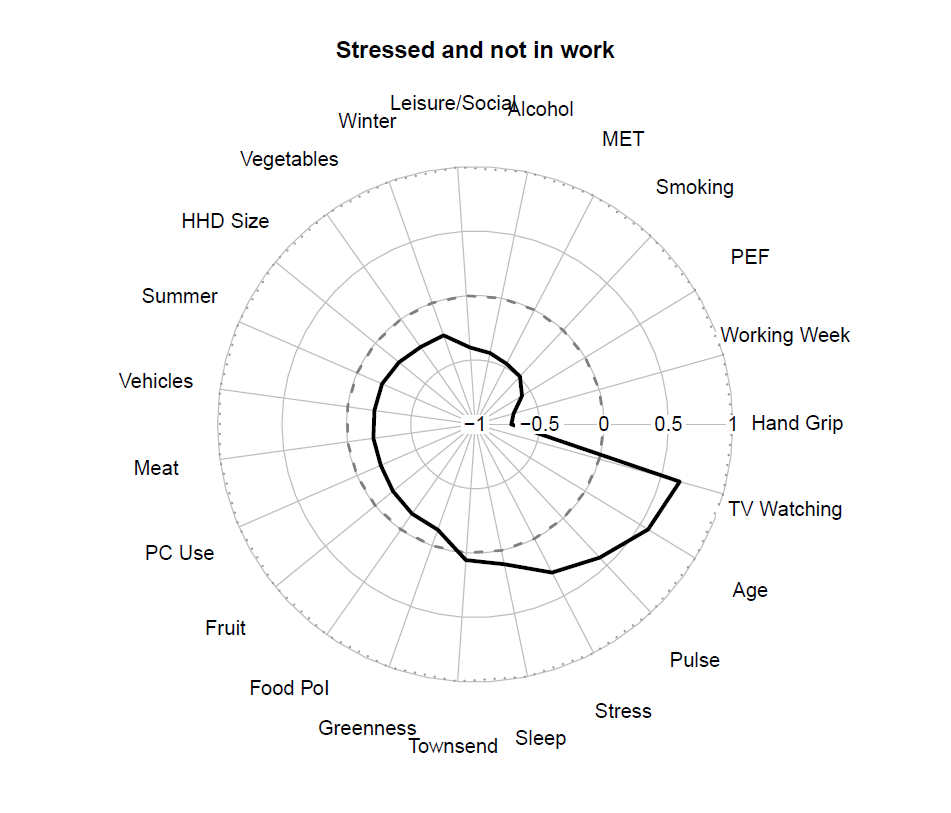


Figure S1f : Classification radar plots for the Stressed and not in work group


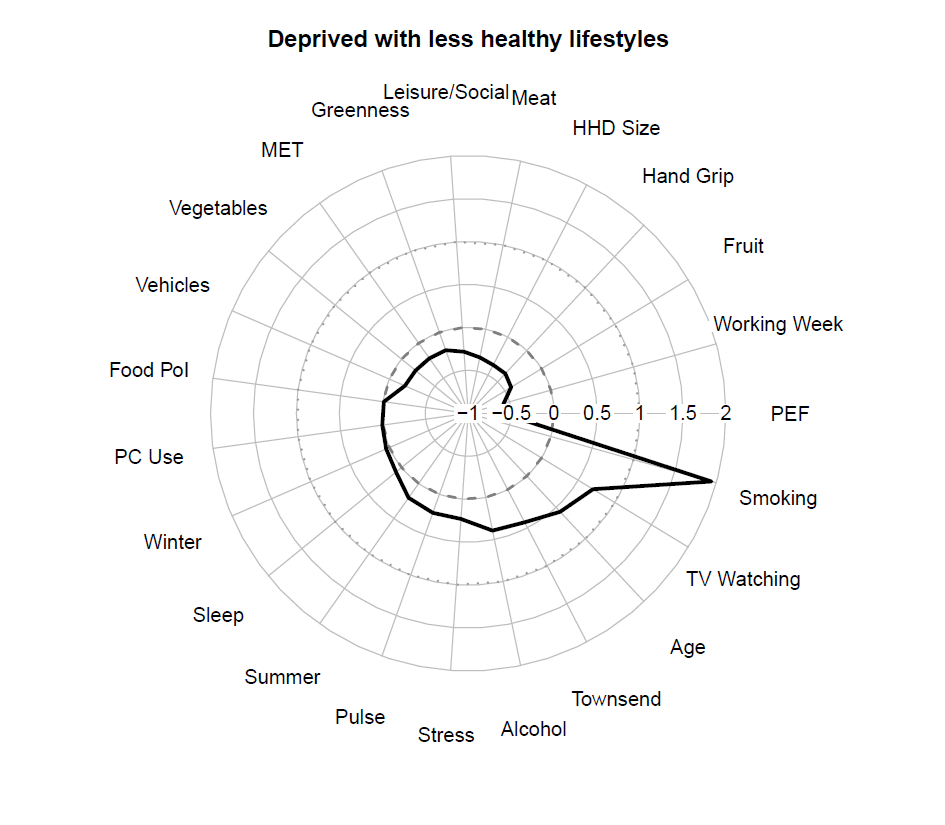


Figure S1g : Classification radar plots for the Deprived with less healthy lifestyles group


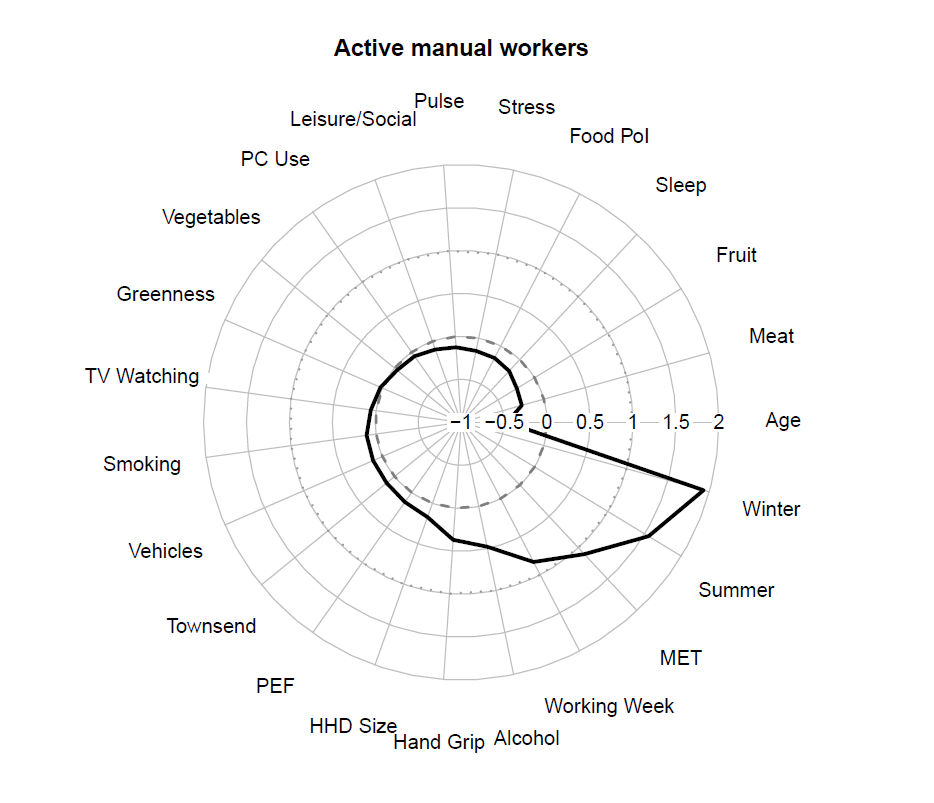


Figure S1h : Classification radar plots for the Active manual workers group
