# Supplementary material for: Correction to: A foresight whole systems obesity classification for the English UK biobank cohort
Source: BMC Public Health. 2022 Apr 11;22:709. doi: 10.1186/s12889-022-13162-4 (PMC8996615; doi:10.1186/s12889-022-13162-4)
Supplement: Supplementary file 1 — Additional file 1: Supplementary file 1. Incorrect version of supplementary 3 as originally published. [file 12889_2022_13162_MOESM1_ESM.docx]

These tables provide percentage counts on how participants in each of the derived classes profiles against a number of key UK Biobank categorical variables. Where the percentage is appreciably more than that for the overall Classification sample it is highlighted in **bold,** and where it is lower it is highlighted in *italic*.

| Table S1 : Distribution of classification by gender (N = 345091) | | |  |  |  |  |  |  |  |
| --- | --- | --- | --- | --- | --- | --- | --- | --- | --- |
| Gender | Younger, urban hard-pressed | Comfortable, fit families | Healthy, active and retirees | Content, rural and retirees | Comfortable professionals | Stressed and not in work | Deprived with less healthy lifestyles | Active manual workers | Full Classification Sample |
| Female | 55.6% | 55.4% | **66.4%** | 53.4% | 54.4% | **69.3%** | *43.5%* | *21.5%* | 54.0% |
| Male | 44.4% | 44.6% | *33.6%* | 46.6% | 45.6% | *30.7%* | **56.5%** | **78.5%** | 46.0% |
| n | 44118 | 39118 | 26721 | 56428 | 41982 | 45273 | 54439 | 37012 | 345091 |
|  |  |  |  |  |  |  |  |  |  |
| Table S2 : Distribution of classification by ethnicity (N = 345091) | | |  |  |  |  |  |  |  |
| Ethnicity | Younger, urban hard-pressed | Comfortable, fit families | Healthy, active and retirees | Content, rural and retirees | Comfortable professionals | Stressed and not in work | Deprived with less healthy lifestyles | Active manual workers | Full Classification Sample |
| White (British/Irish) | *86.0%* | 95.9% | *92.1%* | **99.0%** | **97.5%** | 94.2% | **97.3%** | 94.9% | 94.8% |
| Mixed | **1.4%** | 0.7% | 0.6% | 0.2% | 0.4% | 0.4% | 0.6% | 0.7% | 0.6% |
| Asian (incl. Chinese) | **5.1%** | 1.9% | **3.1%** | *0.3%* | *1.0%* | **3.4%** | *0.7%* | 1.5% | 2.1% |
| Black | **4.7%** | 0.8% | **2.4%** | *0.1%* | *0.5%* | 1.0% | *0.6%* | 2.0% | 1.4% |
| Other | **2.3%** | 0.6% | **1.5%** | 0.2% | 0.4% | 0.7% | 0.5% | 0.6% | 0.8% |
| PNA/DK | 0.4% | 0.2% | 0.4% | 0.2% | 0.2% | 0.2% | 0.3% | 0.3% | 0.3% |
| n | 44118 | 39118 | 26721 | 56428 | 41982 | 45273 | 54439 | 37012 | 345091 |
|  |  |  |  |  |  |  |  |  |  |

| Table S3 : Distribution of classification by BMI category and mean (N = 345091) | | | |  |  |  |  |  |  |
| --- | --- | --- | --- | --- | --- | --- | --- | --- | --- |
| BMI | Younger, urban hard-pressed | Comfortable, fit families | Healthy, active and retirees | Content, rural and retirees | Comfortable professionals | Stressed and not in work | Deprived with less healthy lifestyles | Active manual workers | Full Classification Sample |
| Underweight | 0.7% | 0.5% | 0.7% | 0.3% | 0.5% | 0.6% | 0.6% | 0.1% | 0.5% |
| Healthy | **39.6%** | **39.6%** | **37.4%** | 34.9% | 33.6% | *27.5%* | *24.7%* | *24.6%* | 33.5% |
| Overweight | *38.9%* | 41.7% | 41.9% | **46.8%** | 41.9% | *40.1%* | 43.5% | **48.2%** | 42.7% |
| Obese | *20.7%* | *18.2%* | *19.8%* | *17.9%* | 23.9% | **31.4%** | **30.8%** | **27.0%** | 23.0% |
| NA | 0.2% | 0.1% | 0.2% | 0.1% | 0.1% | 0.4% | 0.3% | 0.1% | 0.2% |
| Median BMI | *26.0* | *26.0* | 26.1 | 26.3 | 26.6 | **27.5** | **27.7** | **27.5** | 26.6 |
| Mean BMI | *26.8* | *26.6* | 26.8 | 26.8 | 27.3 | **28.3** | **28.3** | **28.0** | 27.3 |
| n | 44118 | 39118 | 26721 | 56428 | 41982 | 45273 | 54439 | 37012 | 345091 |
|  |  |  |  |  |  |  |  |  |  |
| Table S4 : Distribution of classification by self-rated health (N = 345091) | | |  |  |  |  |  |  |  |
| Overall health | Younger, urban hard-pressed | Comfortable, fit families | Healthy, active and retirees | Content, rural and retirees | Comfortable professionals | Stressed and not in work | Deprived with less healthy lifestyles | Active manual workers | Full Classification Sample |
| Excellent | 18.9% | **22.4%** | **19.6%** | 21.6% | 18.9% | *8.6%* | *7.3%* | 16.1% | 17.3% |
| Good | 58.0% | 61.3% | 61.4% | 64.5% | 61.1% | *54.2%* | *51.0%* | 61.2% | 59.4% |
| Fair | 19.4% | *14.7%* | *16.3%* | *12.6%* | 17.7% | **28.6%** | **32.3%** | 20.5% | 19.5% |
| Poor | 3.3% | *1.4%* | *2.4%* | *1.2%* | 2.1% | **8.2%** | **8.9%** | 2.0% | 3.5% |
| PNA/DK | 0.4% | 0.1% | 0.2% | 0.1% | 0.2% | 0.4% | 0.5% | 0.3% | 0.3% |
| NA | 0.0% | 0.0% | 0.0% | 0.0% | 0.0% | 0.0% | 0.0% | 0.0% | 0.0% |
| n | 44118 | 39118 | 26721 | 56428 | 41982 | 45273 | 54439 | 37012 | 345091 |
|  |  |  |  |  |  |  |  |  |  |

| Table S5 : Distribution of classification by limiting long-term illness (N = 345091) | | | |  |  |  |  |  |  |
| --- | --- | --- | --- | --- | --- | --- | --- | --- | --- |
| Long standing illness | Younger, urban hard-pressed | Comfortable, fit families | Healthy, active and retirees | Content, rural and retirees | Comfortable professionals | Stressed and not in work | Deprived with less healthy lifestyles | Active manual workers | Full Classification Sample |
| No long standing illness | **72.8%** | **80.5%** | 66.0% | 70.3% | **72.9%** | *54.6%* | *51.3%* | **73.4%** | 68.4% |
| Long standing illness | *25.2%* | *18.1%* | 32.0% | 28.1% | *25.4%* | **42.7%** | **45.9%** | *24.7%* | 29.7% |
| PNA/DK | 2.1% | *1.4%* | 2.0% | 1.6% | 1.6% | **2.8%** | **2.8%** | 2.0% | 2.0% |
| NA | 0.0% | 0.0% | 0.0% | 0.0% | 0.0% | 0.0% | 0.0% | 0.0% | 0.0% |
| n | 44118 | 39118 | 26721 | 56428 | 41982 | 45273 | 54439 | 37012 | 345091 |
|  |  |  |  |  |  |  |  |  |  |
| Table S6 : Distribution of classification by doctor diagnosed diabetes (N = 345091) | | | |  |  |  |  |  |  |
| Diabetes | Younger, urban hard-pressed | Comfortable, fit families | Healthy, active and retirees | Content, rural and retirees | Comfortable professionals | Stressed and not in work | Deprived with less healthy lifestyles | Active manual workers | Full Classification Sample |
| No Diabetes | 96.0% | **98.1%** | 94.5% | 95.9% | 96.6% | *91.3%* | *90.4%* | 95.7% | 95.0% |
| Diabetes | 3.7% | *1.8%* | 5.3% | 4.0% | 3.3% | **8.3%** | 9.3% | 4.0% | 4.8% |
| PNA/DK | 0.0% | 0.0% | 0.0% | 0.0% | 0.0% | 0.0% | 0.0% | 0.0% | 0.0% |
| NA | 0.0% | 0.0% | 0.0% | 0.0% | 0.0% | 0.0% | 0.0% | 0.0% | 0.0% |
| n | 44118 | 39118 | 26721 | 56428 | 41982 | 45273 | 54439 | 37012 | 345091 |
|  |  |  |  |  |  |  |  |  |  |
| Table S7 : Distribution of classification by doctor diagnosed cancer (N = 345091) | | | |  |  |  |  |  |  |
| Cancer | Younger, urban hard-pressed | Comfortable, fit families | Healthy, active and retirees | Content, rural and retirees | Comfortable professionals | Stressed and not in work | Deprived with less healthy lifestyles | Active manual workers | Full Classification Sample |
| No Cancer | **94.6%** | **95.9%** | *89.8%* | *90.2%* | 93.7% | *89.6%* | *89.7%* | 95.5% | 92.4% |
| Cancer | *5.2%* | *4.0%* | **9.9%** | **9.6%** | 6.1% | **10.0%** | **9.8%** | 4.3% | 7.4% |
| PNA/DK | 0.0% | 0.0% | 0.0% | 0.0% | 0.0% | 0.0% | 0.0% | 0.0% | 0.0% |
| NA | 0.0% | 0.0% | 0.0% | 0.0% | 0.0% | 0.0% | 0.0% | 0.0% | 0.0% |
| n | 44118 | 39118 | 26721 | 56428 | 41982 | 45273 | 54439 | 37012 | 345091 |
|  |  |  |  |  |  |  |  |  |  |
| Table S8 : Distribution of classification by doctor diagnosed other illness (N = 345091) | | | |  |  |  |  |  |  |
| Other illness | Younger, urban hard-pressed | Comfortable, fit families | Healthy, active and retirees | Content, rural and retirees | Comfortable professionals | Stressed and not in work | Deprived with less healthy lifestyles | Active manual workers | Full Classification Sample |
| None | 81.7% | **86.9%** | 77.0% | 79.7% | 82.3% | *70.9%* | *71.2%* | **85.7%** | 79.6% |
| Some | 17.0% | *12.1%* | 21.4% | 19.2% | 16.6% | **26.6%** | **26.4%** | *13.1%* | 18.9% |
| PNA/DK | 0.0% | 0.0% | 0.0% | 0.0% | 0.0% | 0.0% | 0.0% | 0.0% | 0.0% |
| NA | 0.0% | 0.0% | 0.0% | 0.0% | 0.0% | 0.0% | 0.0% | 0.0% | 0.0% |
| n | 44118 | 39118 | 26721 | 56428 | 41982 | 45273 | 54439 | 37012 | 345091 |
|  |  |  |  |  |  |  |  |  |  |
| Table S9 : Distribution of classification by experience of shortness of breath (N = 345091) | | | | |  |  |  |  |  |
| Shortness in breath on walking on level ground | Younger, urban hard-pressed | Comfortable, fit families | Healthy, active and retirees | Content, rural and retirees | Comfortable professionals | Stressed and not in work | Deprived with less healthy lifestyles | Active manual workers | Full Classification Sample |
| No | **41.2%** | 31.6% | **42.5%** | 33.5% | *30.9%* | 32.3% | *30.7%* | 35.0% | 34.5% |
| Yes | 3.6% | *1.6%* | 3.5% | *1.8%* | 2.6% | **7.5%** | **7.7%** | 2.5% | 3.7% |
| PNA/DK | 1.0% | 0.4% | 0.5% | 0.2% | 0.5% | **1.2%** | **1.1%** | 0.6% | 0.7% |
| NA | 54.2% | 66.4% | 53.5% | 64.4% | **66.0%** | 58.9% | 60.5% | 62.0% | 61.1% |
| n | 44118 | 39118 | 26721 | 56428 | 41982 | 45273 | 54439 | 37012 | 345091 |
|  |  |  |  |  |  |  |  |  |  |

| Table S10 : Distribution of classification by employment status (N = 345091) | | | |  |  |  |  |  |  |
| --- | --- | --- | --- | --- | --- | --- | --- | --- | --- |
| Employment | Younger, urban hard-pressed | Comfortable, fit families | Healthy, active and retirees | Content, rural and retirees | Comfortable professionals | Stressed and not in work | Deprived with less healthy lifestyles | Active manual workers | Full Classification Sample |
| In paid employment or self-employed | **86.2%** | **92.0%** | *31.9%* | *22.3%* | **95.6%** | *27.6%* | *30.9%* | **90.8%** | 59.0% |
| Retired | *4.2%* | *1.3%* | **58.8%** | **71.6%** | *2.8%* | **57.9%** | **53.5%** | *5.3%* | 32.7% |
| Looking after home and/or family | 3.6% | **4.6%** | 2.8% | 2.6% | 0.4% | **4.0%** | 2.2% | 1.1% | 2.8% |
| Unable to work because of sickness/disability | 1.8% | *0.5%* | 2.3% | *0.8%* | *0.4%* | **6.3%** | **8.3%** | *0.8%* | 2.5% |
| Unemployed | **2.3%** | *0.7%* | 1.8% | 1.0% | *0.4%* | **2.3%** | **3.5%** | 1.2% | 1.6% |
| Doing unpaid or voluntary work | 0.6% | 0.2% | **0.9%** | 0.7% | 0.2% | 0.5% | 0.4% | 0.1% | 0.5% |
| Full or part-time student | 0.7% | 0.4% | 0.3% | 0.1% | 0.1% | 0.1% | 0.1% | 0.1% | 0.2% |
| PNA/DK | 0.7% | 0.3% | **1.2%** | 0.9% | 0.2% | **1.1%** | 0.9% | 0.4% | 0.7% |
| NA | 0.0% | 0.0% | 0.0% | 0.0% | 0.0% | 0.0% | 0.0% | 0.0% | 0.0% |
| n | 44118 | 39118 | 26721 | 56428 | 41982 | 45273 | 54439 | 37012 | 345091 |
|  |  |  |  |  |  |  |  |  |  |
| Table S11 : Distribution of classification by occupation (N = 345091) | | |  |  |  |  |  |  |  |
| Job/Occupation code | Younger, urban hard-pressed | Comfortable, fit families | Healthy, active and retirees | Content, rural and retirees | Comfortable professionals | Stressed and not in work | Deprived with less healthy lifestyles | Active manual workers | Full Classification Sample |
| Managers and senior officials | 15.4% | **19.8%** | *5.9%* | *5.8%* | **21.9%** | *4.0%* | *6.4%* | 12.8% | 11.7% |
| Professional occupations | **24.9%** | **23.8%** | *12.1%* | *10.1%* | **25.5%** | *7.7%* | *6.3%* | *8.5%* | 15.6% |
| Associate professional & technical occupations | **17.3%** | **17.0%** | 9.0% | *5.9%* | **16.0%** | *5.6%* | *5.8%* | 11.2% | 11.1% |
| Administrative and secretarial occupations | **12.0%** | **13.2%** | 9.1% | *6.1%* | **15.7%** | 11.2% | *7.6%* | *5.0%* | 10.3% |
| Skilled trades occupations | 3.5% | 4.3% | *2.7%* | *2.5%* | 4.4% | *2.0%* | 4.2% | **21.8%** | 4.8% |
| Personal service occupations | 4.4% | **5.2%** | 4.0% | *1.9%* | 3.0% | 3.7% | 3.3% | **5.3%** | 3.8% |
| Sales and customer service occupations | 2.2% | 2.2% | 2.1% | *1.3%* | 2.0% | 3.3% | 2.6% | 2.7% | 2.2% |
| Process, plant and machine operatives | 1.9% | *1.9%* | 1.6% | *1.1%* | 2.4% | *1.8%* | 3.5% | **12.8%** | 2.8% |
| Elementary occupations | 3.3% | 2.0% | 2.6% | *1.2%* | *1.7%* | 3.4% | 4.1% | **8.7%** | 3.0% |
| Other | 2.8% | 3.1% | 3.2% | 2.1% | **3.7%** | 2.3% | 2.4% | 3.4% | 2.8% |
| NA | 12.3% | 7.7% | **47.7%** | **62.0%** | 3.7% | **55.2%** | **54.1%** | 7.7% | 31.9% |
| n | 44118 | 39118 | 26721 | 56428 | 41982 | 45273 | 54439 | 37012 | 345091 |
|  |  |  |  |  |  |  |  |  |  |
| Table S12 : Distribution of classification by job involves walking or standing (N = 203730) | | | | |  |  |  |  |  |
| Walking or standing | Younger, urban hard-pressed | Comfortable, fit families | Healthy, active and retirees | Content, rural and retirees | Comfortable professionals | Stressed and not in work | Deprived with less healthy lifestyles | Active manual workers | Full Classification Sample |
| Never/rarely | **43.9%** | 41.3% | *28.2%* | 30.9% | **45.1%** | 33.1% | *28.1%* | *11.9%* | 36.4% |
| Sometimes | 30.5% | 32.0% | 30.5% | 32.2% | 33.0% | 28.2% | 28.4% | *25.2%* | 30.6% |
| Usually | 12.1% | 13.3% | **16.7%** | **17.2%** | *11.3%* | 14.8% | 15.5% | **22.6%** | 14.4% |
| Always | *13.5%* | *13.4%* | **24.5%** | 19.6% | *10.6%* | **23.7%** | **28.0%** | **40.2%** | 18.5% |
| PNA/DK | 0.0% | 0.0% | 0.1% | 0.1% | 0.0% | 0.1% | 0.1% | 0.1% | 0.0% |
| *NA* |  |  |  |  |  |  |  |  |  |
| n | 38019 | 12478 | 24271 | 12576 | 11596 | 43267 | 50072 | 11451 | 203730 |
|  |  |  |  |  |  |  |  |  |  |
| Table S13 : Distribution of classification by job involves heavy lifting or physical tasks (N = 203730) | | | | |  |  |  |  |  |
| Heavy or physical | Younger, urban hard-pressed | Comfortable, fit families | Healthy, active and retirees | Content, rural and retirees | Comfortable professionals | Stressed and not in work | Deprived with less healthy lifestyles | Active manual workers | Full Classification Sample |
| Never/rarely | **73.9%** | **73.0%** | 62.0% | 67.0% | **76.5%** | 65.7% | *55.5%* | *27.7%* | 66.1% |
| Sometimes | 17.7% | *18.8%* | 23.4% | 22.4% | *17.2%* | 22.4% | **26.9%** | **31.6%** | 21.0% |
| Usually | *4.4%* | *4.6%* | 7.4% | 6.3% | *3.7%* | 6.0% | **8.9%** | **18.5%** | 6.6% |
| Always | *3.9%* | *3.5%* | 7.0% | 4.3% | *2.5%* | 5.8% | **8.6%** | **22.1%** | 6.3% |
| PNA/DK | 0.0% | 0.0% | 0.1% | 0.0% | 0.0% | 0.1% | 0.1% | 0.1% | 0.0% |
| *NA* |  |  |  |  |  |  |  |  |  |
| n | 38019 | 12478 | 24271 | 12576 | 11596 | 43267 | 50072 | 11451 | 203730 |
|  |  |  |  |  |  |  |  |  |  |

| Table S14 : Distribution of classification by job involves shift work (N = 203730) | | | |  |  |  |  |  |  |
| --- | --- | --- | --- | --- | --- | --- | --- | --- | --- |
| Shift work | Younger, urban hard-pressed | Comfortable, fit families | Healthy, active and retirees | Content, rural and retirees | Comfortable professionals | Stressed and not in work | Deprived with less healthy lifestyles | Active manual workers | Full Classification Sample |
| Never/rarely | 83.5% | 86.1% | 82.4% | **89.5%** | **87.8%** | 84.7% | *78.5%* | *70.3%* | 83.6% |
| Sometimes | 7.3% | 6.2% | 7.4% | *4.9%* | *5.8%* | *5.3%* | 8.1% | **12.4%** | 7.1% |
| Usually | 2.0% | *1.6%* | 2.5% | *1.5%* | 1.4% | 2.4% | 2.8% | **3.1%** | 2.0% |
| Always | 6.9% | 6.0% | 7.5% | *4.0%* | *4.9%* | 7.3% | **10.5%** | **14.1%** | 7.2% |
| PNA/DK | 0.2% | 0.1% | 0.2% | 0.1% | 0.1% | 0.2% | 0.1% | 0.1% | 0.1% |
| *NA* |  |  |  |  |  |  |  |  |  |
| n | 38019 | 12478 | 24271 | 12576 | 11596 | 43267 | 50072 | 11451 | 203730 |
|  |  |  |  |  |  |  |  |  |  |
| Table S15 : Distribution of classification by educational qualification (N = 345091) | | | |  |  |  |  |  |  |
| Education | Younger, urban hard-pressed | Comfortable, fit families | Healthy, active and retirees | Content, rural and retirees | Comfortable professionals | Stressed and not in work | Deprived with less healthy lifestyles | Active manual workers | Full Classification Sample |
| College or University degree | **55.0%** | **43.7%** | 33.3% | 31.9% | **42.1%** | *19.5%* | *15.7%* | *16.2%* | 33.7% |
| A levels/AS levels or equivalent | 12.2% | **15.8%** | 10.4% | 11.4% | 14.1% | 9.6% | *8.4%* | *8.3%* | 11.7% |
| O levels/GCSEs or equivalent | *15.0%* | 22.9% | 20.1% | 23.8% | 22.0% | 24.5% | 22.6% | **24.8%** | 21.9% |
| CSEs or equivalent | 5.3% | 7.5% | *3.4%* | 2.8% | 5.1% | 5.2% | 5.0% | **13.8%** | 5.6% |
| NVQ or HND or HNC or equivalent | 4.1% | 4.4% | 6.1% | 6.9% | 6.4% | 5.9% | **9.1%** | **12.3%** | 6.5% |
| Other professional qualifications eg: nursing, teaching | 3.1% | *3.0%* | 6.6% | 7.4% | 4.7% | 5.9% | 5.5% | 4.5% | 5.1% |
| PNA/DK | *5.3%* | *2.6%* | **20.0%** | 16.0% | *5.7%* | **29.4%** | **33.9%** | **19.9%** | 15.5% |
| NA | 0.0% | 0.0% | 0.0% | 0.0% | 0.0% | 0.0% | 0.0% | 0.0% | 0.0% |
| n | 44118 | 39118 | 26721 | 56428 | 41982 | 45273 | 54439 | 37012 | 345091 |
|  |  |  |  |  |  |  |  |  |  |

| Table S16 : Distribution of classification by work satisfaction (N = 96278) | | | |  |  |  |  |  |  |
| --- | --- | --- | --- | --- | --- | --- | --- | --- | --- |
| Work satisfaction | Younger, urban hard-pressed | Comfortable, fit families | Healthy, active and retirees | Content, rural and retirees | Comfortable professionals | Stressed and not in work | Deprived with less healthy lifestyles | Active manual workers | Full Classification Sample |
| Extremely happy | *6.8%* | 7.1% | **11.2%** | **11.0%** | 7.2% | *6.9%* | 9.0% | 9.8% | 8.3% |
| Very happy | 32.7% | *33.2%* | **42.3%** | **49.0%** | *32.7%* | 35.2% | 34.8% | 34.4% | 36.0% |
| Moderately happy | 47.3% | **48.1%** | *40.4%* | *36.5%* | 47.2% | **48.5%** | 45.7% | 45.3% | 45.4% |
| Moderately unhappy | **9.3%** | 8.6% | *3.4%* | *2.3%* | **9.6%** | *5.6%* | 6.4% | 7.1% | 7.1% |
| Very unhappy | **2.4%** | 2.0% | 1.0% | *0.5%* | **2.2%** | 1.3% | 1.8% | 2.1% | 1.8% |
| Extremely unhappy | 1.1% | 0.8% | 0.6% | 0.1% | 1.0% | 0.7% | 1.0% | 1.1% | 0.8% |
| I am not employed |  |  |  |  |  |  |  |  |  |
| PNA/DK | 0.5% | 0.2% | 1.1% | 0.6% | 0.1% | 1.8% | 1.3% | 0.3% | 0.6% |
| NA |  |  |  |  |  |  |  |  |  |
| n | 18675 | 10451 | 9600 | 9421 | 8412 | 14948 | 17477 | 7294 | 96278 |
|  |  |  |  |  |  |  |  |  |  |
| Table S17 : Distribution of classification by health satisfaction (N = 134212) | | | |  |  |  |  |  |  |
| Health satisfaction | Younger, urban hard-pressed | Comfortable, fit families | Healthy, active and retirees | Content, rural and retirees | Comfortable professionals | Stressed and not in work | Deprived with less healthy lifestyles | Active manual workers | Full Classification Sample |
| Extremely happy | 5.0% | 5.8% | **6.8%** | **6.5%** | 4.7% | *3.0%* | *3.0%* | **6.4%** | 5.2% |
| Very happy | 32.7% | **37.5%** | 36.8% | **40.2%** | 33.8% | *23.9%* | *22.0%* | 35.2% | 33.0% |
| Moderately happy | 47.8% | *46.4%* | *46.9%* | *46.8%* | 49.5% | **53.1%** | **54.8%** | 48.8% | 49.1% |
| Moderately unhappy | 10.6% | 8.1% | *6.8%* | *5.0%* | 9.6% | **12.3%** | **13.6%** | 7.3% | 9.1% |
| Very unhappy | 2.5% | *1.5%* | *1.7%* | *0.9%* | 1.8% | **4.8%** | **4.3%** | 1.4% | 2.4% |
| Extremely unhappy | 1.0% | *0.5%* | 0.7% | *0.3%* | 0.5% | **2.1%** | **1.8%** | 0.5% | 0.9% |
| PNA/DK | 0.4% | *0.2%* | 0.4% | 0.2% | 0.1% | 0.8% | 0.5% | 0.3% | 0.4% |
| NA |  |  |  |  |  |  |  |  |  |
| n | 20196 | 18205 | 10165 | 20089 | 17240 | 15409 | 18299 | 14609 | 134212 |
|  |  |  |  |  |  |  |  |  |  |

| Table S18 : Distribution of classification by assessment centre (N = 345091) | | | |  |  |  |  |  |  |
| --- | --- | --- | --- | --- | --- | --- | --- | --- | --- |
| Location of assessment centre | Younger, urban hard-pressed | Comfortable, fit families | Healthy, active and retirees | Content, rural and retirees | Comfortable professionals | Stressed and not in work | Deprived with less healthy lifestyles | Active manual workers | Full Classification Sample |
| Newcastle | *4.5%* | 7.5% | 7.4% | 7.1% | 7.7% | **9.5%** | **9.2%** | **9.0%** | 7.6% |
| Middlesbrough | *1.5%* | 5.3% | *3.6%* | **7.0%** | 4.9% | 5.2% | 5.6% | 6.4% | 5.0% |
| Manchester | **4.7%** | 3.0% | 3.8% | *1.7%* | 3.0% | *2.3%* | 3.5% | 3.4% | 3.1% |
| Liverpool | *5.6%* | 6.5% | 8.3% | 7.0% | 6.6% | **9.1%** | **9.9%** | 8.3% | 7.5% |
| Bury | *2.6%* | 6.6% | *4.8%* | 7.1% | **7.3%** | 6.9% | **7.8%** | 7.1% | 6.2% |
| Leeds | *4.7%* | **11.9%** | 7.6% | 11.3% | **11.6%** | 10.3% | 10.6% | 10.4% | 9.9% |
| Sheffield | *4.2%* | 7.2% | *5.8%* | **8.9%** | 7.1% | 7.3% | 7.1% | 7.8% | 7.0% |
| Nottingham | *4.9%* | 8.4% | 7.2% | **10.1%** | 8.5% | 8.5% | 8.1% | 8.3% | 8.1% |
| Stoke | *1.1%* | 4.5% | *2.3%* | **5.6%** | 4.5% | 4.1% | 3.6% | 5.1% | 3.9% |
| Birmingham | 7.1% | 4.3% | **8.0%** | 2.8% | 5.2% | **7.7%** | 7.1% | 5.1% | 5.7% |
| Oxford | *1.5%* | **5.1%** | 2.0% | 4.2% | **4.5%** | 2.0% | *1.9%* | 2.7% | 3.2% |
| Bristol | 9.3% | 12.0% | 8.5% | **12.5%** | 10.8% | 8.7% | *7.7%* | 10.0% | 10.2% |
| Reading | *2.3%* | **10.9%** | *4.2%* | **10.5%** | **10.4%** | 5.6% | *4.3%* | 5.6% | 7.1% |
| London (Barts) | **16.1%** | *0.0%* | 3.6% | *0.0%* | *0.1%* | 0.5% | *1.9%* | *0.8%* | 2.8% |
| Hounslow | **14.9%** | *3.5%* | **12.1%** | *1.7%* | *4.5%* | 6.8% | 6.3% | *5.3%* | 6.6% |
| Croydon | **14.9%** | *3.4%* | **10.6%** | *2.3%* | *3.2%* | 5.4% | 5.6% | *4.6%* | 6.0% |
| n | 44118 | 39118 | 26721 | 56428 | 41982 | 45273 | 54439 | 37012 | 345091 |

**Abbreviations:**

N Number of participants responding

n number of responding participants in the class

PNA/DK Prefer Not to Answer or Don’t Know

NA Not Available

BMI Body Mass Index

CSE Certificate of Secondary Education

NVQ National Vocational Qualification

HND Higher National Diploma

HNC Higher National Certificate
